# Protective Effects of Squid Ink Extract Towards Hemopoietic Injuries Induced by Cyclophosphamine

**DOI:** 10.3390/md7010009

**Published:** 2009-01-14

**Authors:** Jie-Ping Zhong, Guang Wang, Jiang-Hua Shang, Jiang-Qiu Pan, Kun Li, Yan Huang, Hua-Zhong Liu

**Affiliations:** 1 Modern Biochemistry Center, Guangdong Ocean University, Zhanjiang 524088, P.R.China; 2 College of Food Science & Technology, Guangdong Ocean University, Zhanjiang 524088, P.R.China; 3 Buffalo Research Institute, Chinese Academy of Agricultural Sciences, Nanning 530001, P.R. China

**Keywords:** Squid ink, cyclophosphamine, hemopoiesis, mice

## Abstract

To investigate the protective effects of squid ink in chemotherapy, BALB/c mice were used as animal models of injuries induced by cyclophosphamine, a well known chemotherapeutic drug. The mice were randomly divided into five groups with the same number of males and females in each group. At the end of the experiment, animals were sacrificed to investigate organ indexes and antioxidant ability of the spleen, peripheral blood profile and quantities of bone marrow nucleated cells. Results showed that the hemopoietic function of mice was injured by cyclophosphamine, as indicated by decreases of contents of erythrocytes, leukocytes, hemoglobin and bone marrow nucleated cells (P<0.01), while  platelets were not affected (P>0.05), as well as modification of organ indexes (P<0.05) and spleen antioxidant ability (P<0.05 or P<0.01), whereas sepia extract markedly increased the levels of erythrocytes, leukocytes, hemoglobin and bone marrow nucleated cells (P<0.01), but not platelets (P>0.05), and reversed the effects of cyclophosphamine on organ indexes and antioxidant ability of spleen (P<0.01 or P<0.05). In addition, squid ink extract did not change marrow hemopoiesis but improved the antioxidant ability of spleen in the animals. The data suggest that squid ink extract can protect the hemopoietic system from chemotherapeutic injury and could be employed to develop cell-protective drugs for use in clinical treatment of tumours.

## 1. Introduction

The cuttlefish *Sepia officinalis* relies for defense on the ejection of a dark ink (commonly called sepia) which consists of a suspension of melanin granules in a viscous colorless medium. At the end of the maturation process, ink gland cells of the digestive tract in the mantle cavity degenerate and shed their contents into the ink sac, acting as a reservoir of the exhausted material. Both production and ejection of the ink appear to be regulated by the glutamate/nitric oxide/cGMP signaling pathway which is localized in the ink gland [1,2] as well as in the central nervous system and in certain neural pathways controlling the ink sac sphincters and wall muscle [3]. Besides large amounts of melanin, the ink also contains proteins, lipids, glycosaminoglycans, etc.

Cuttlefish ink is a traditional Chinese medicine listed in the *Compendium of Materia Medica* compiled by Shizhen Li, a famous doctor at the time of the Ming Dynasty, and first employed to treat heart pain, but modern clinical medicine has proven that it is a good hemostasic medicine that also provides significant curative effects in gynecology, surgery, and so on. Background research has shown that squid ink possesses a wide range of biological roles as it not only inhibits the activity of plasmin to promote thromboxane and elevates immunological competence to kill cancer cells to thus exert its anti-tumour effects [4], but it also has leukocyte-number elevating [5–7], anti-oxidant [8], anti-radiation [9], anti-retrovirus [10], and anti-bacterial properties [11–13]. Presently, there is little information about the protective effects of sepia ink on hemopoietic injury. It is documented that sepia ink could induce marrow cells to produce multiple colony stimulating factors promoting marrow cell proliferation to form colonies *in vitro* [6,14], which results in an elevation of leukocyte numbers decreased by chemotherapy. The results have been supported by our recent investigation [15], showing that squid ink could heighten quantities of leukocytes and bone marrow nucleated cells reduced by cyclophosphamine. In addition, Lei *et al.* [9] showed that cuttlefish ink could protect against hemopoietic injury induced by radiation.

Tumours are one of three main causes of death faced by humans, and although many new ways have been developed to treat the disease, chemotherapy is still the main clinical treatment method used. It is well known that chemotherapy leads to body injury as the drugs kill tumor cells, so the study and development of cell-protective drugs is urgent. Because of the extensive development seen in recent years of medicines from marine sources, the search for effective cell-protective drugs from the sea has become an important activity. Based on the current information, the aim of the present work was to investigate the protective effects of squid ink extract on hemopoietic injury induced by chemotherapeutics and to develop a theoretical foundation for the potential clinical application of squid ink.

## 2. Materials and Methods

### 2.1 Preparation of squid ink extract

Fresh cuttlefish (*Sepia officinalis*) were purchased directly from a fishmonger and rapidly transferred to the laboratory where they were dissected and the ink was collected in a mortar. The ink was diluted immediately with an equal volume of phosphate buffered saline (PBS, pH 6.8) and ground sufficiently followed by ultrasonication. The admixture was collected in centrifugation tubes and centrifuged at a speed of 15,000 g for 20 min at 4°C. The supernatant was collected immediately, freeze-dried and stored  at −80°C. The sample was dissolved in normal saline and diluted to the appropriate concentration, and sterilized through 0.22 μm filters before administration to animals.

### 2.2 Animals and design of the experiment

Healthy BLAB/c mice (weighing 20±2 g, SPF, purchased from the Laboratory Animal Center of Guangdong Medical College) were divided randomly into five groups of 20 mice per group with 10 male and 10 female anmals in each group. Mice in the control group (Group A) were treated orally with normal saline and injected abdominally with normal saline; mice in the positive control group (Group B) were administered squid ink extract (1.2 g/kg body weight) orally and injected abdominally with normal saline; mice in the model group (Group C) were administered normal saline orally and injected abdominally with cyclophosphamine (200 mg/kg body weight); mice in the low-dose group (Group D) were administered squid ink extract (1.2 g/kg body weight) orally and injected abdominally with cyclophosphamine (200 mg/kg body weight), and finally mice in the high-dose group (Group E) were administered squid ink extract (2.4 g/kg body weight) orally and injected abdominally with cyclophosphamine (200 mg/kg body weight). Mice were administered normal saline or the indicated concentration of ink extract orally for ten days, followed by injection with normal saline or cyclophosphamine for three days, and then, oral administration with saline or ink extract for another ten days. On the next day, all animals were sacrificed to examine peripheral blood profile, bone marrow nucleated cells count, DNA cotent of bone marrow cells as well as organ-index and antioxidant ability of spleen.

### 2.3 Peripheral blood profile

Blood was obtaned from eyes and used to examine quantities of blood cells, including erythrocytes, leukocytes and platelets, and levels of hemoglobin with an automatic hematocyte counter.

### 2.4 Bone marrow nucleated cells counting [9]

Bone marrow cells were collected from mouse femora with normal saline (1 mL), collected in 1.5 mL Eppendorf tubes, and dyed with hexamethylpararosaniline dissolved in 2% acetic acid. Then a cell counter was employed to count bone marrow nucleated cells.

### 2.5 DNA contents detection of bone marrow [16]

Other mouse femora was used to prepare DNA. Bone marrow cells were collected in a centrifuge tube with 0.005 mol/L CaCl_2_ solution (10 mL), stored at 4°C for 30min, and centrifuged at the speed of 2,500 rpm for 15min. Sediment was harvested and dissolved in 1.2 mol/L HClO_4_ solution (5 mL), and then heated at 90°C for 15min. The mixture was cooled with ice water and centrifuged at 3,000 rpm for 10 min. The supernatant was used to detect optical density value at the wavelength of 268 nm.

### 2.6 Spleen organ index

The spleen of the mice were removed and weighed immediately after sacrifice, the organ index was calculated and denoted as the ratio of organ weight to body weight, milligram per gram (mg/g).

### 2.7 Antioxidant ability of spleen

Spleens were homogenated and centrifuged at 4°C, the supernatant was prepared to detect activities of superoxide dismutase (SOD), catalase (CAT) and glutathione peroxidase (GSH-Px) as well as the content of malondialdehyde (MDA). The parameters were determined with kits (catalogue No.: 20080619) developed by the Bioengineering Institute of Nanjing Jiancheng, P.R. China, according to the manufacturer’s protocol.

### 2.8 Statistical analysis

Data were summarized as means ± standard deviation. Statistical analysis was carried out using SPSS11.0 software (SPSS, Chicago, USA) and one-way ANOVA was used to determine significant differences between groups. A probability value less than 0.05 (*P<*0.05) was considered to be statistically significant.

## 3. Results

### 3.1 Peripheral blood profile

**Figure 1:**
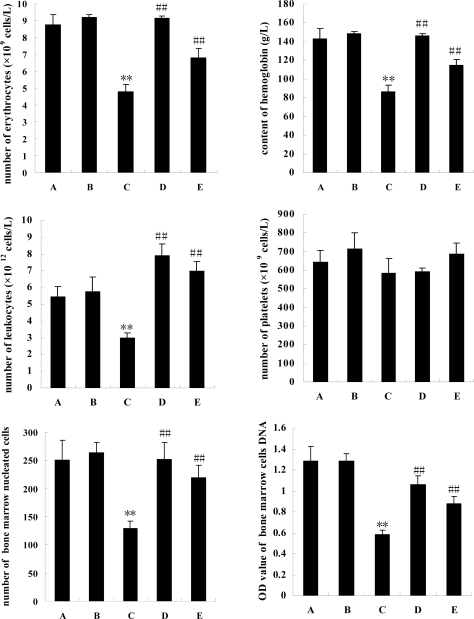
The effects of squid ink extract on peripheral blood profile and bone marrow nucleated cells in mice induced by cyclophosphamine. Bars indicate means±standard deviations. ** indicates that there was a extremely significant difference (P<0.01) between model mice (group C) and control mice (group A), ## indicates a difference (P<0.01) between low-dose group (group D) or high-dose group(group E) and model mice (group C). Group B represents positive control group.

In Figure 1 the data shows that cyclophosphamine impaired contents of erythrocytes, leukocytes and hemoglobin in peripheral blood (P<0.01), but not platelets (P>0.05). Comparison with model mice with injuries induced by cyclophosphamine (group C), squid ink significantly elevated their contents of erythrocytes, leukocytes and hemoglobin in peripheral blood (P<0.01), but the marine drug also failed to change the quantities of platelets in two experimental mice groups (groups D or E, P>0.05).

### 3.2 Numbers of bone marrow cells and its DNA contents

To investigate the effects of squid ink on hemopoietic function of mice, bone marrow nucleated cells were counted (Figure 1). Cyclophosphamine severely impaired the amount of bone marrow nucleated cells (P<0.01), whereas squid ink extract reversed the inhibitory effects and heightened the contents of the cells (P<0.01). To further verify the outcomes, DNA contents of bone marrow cells were examined, and the same results were obtained.

### 3.3 Organ index of spleen in mice

**Figure 2:**
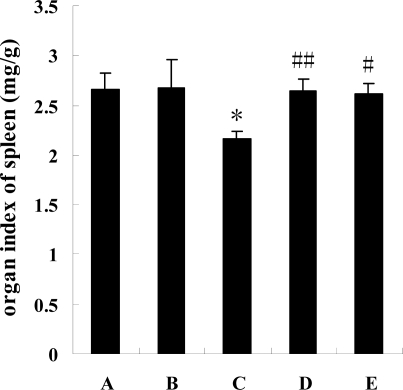
The effects of squid ink on organ index of spleen in mice induced by cyclophosphamine. Bars indicate means±standard deviations. * indicates that there was a significant difference (P<0.05) between model mice (group C) and control mice (group A), ## indicates a difference (P<0.01) between low-dose group (group D) and model mice (group C), # indicates a difference (P<0.05) between high-dose group (group E) and model mice. Group B represents positive control group.

In Figure 2 it is shown that the organs in the model mice were affected by cyclophosphamine, and the organ indexes of their spleens were significantly synchronously downregulated (P<0.05) by the chemotherapeutic treatment. The figure shows that the parameters in the experimental mice (groups D and E) showed an obvious elevation (P<0.01 or P<0.05) compared to model group mice.

### 3.4 Antioxidant ability of the spleen

**Figure 3:**
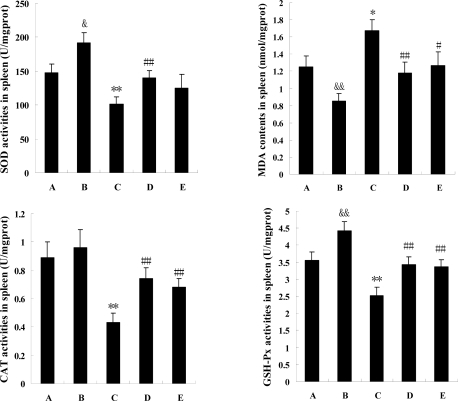
The effects of squid ink on antioxidant ability of spleen in mice induced by cyclophosphamine. Bars indicate means±standard deviations. ^&^*P*<0.05, or ^&&^*P*<0.01, vs the value of control mice (group A). *P<0.05, or **P<0.01, vs the value of control mice (group A). ^#^*P*<0.05, or ^##^*P*<0.01, vs the value of model mice (group C). Group B represents positive control group.

Data (Figure 3) indicated that four parameters of spleen antioxidant ability were all modified markedly by cyclophosphamine (P<0.05 or P<0.01), whereas,  the changes were all reversed by squid ink extract and low dosage squid ink extract redressed significantly the alterations induced by the chemotherapeutics (P<0.01). Effects of the high dosage of marine drug on the changes were also observed, but the results were diverse, as the medicine changed significantly activities of CAT and GSH-Px (P<0.01) as well as content of MDA (P<0.05), and did not alter the activity of SOD (P>0.05). In addition, this experiment revealed that squid ink extract changed the antioxidant ability of the spleen in mice, as the SOD and GSH-Px activities in the spleens from the positive control mice were higher than those in spleens from control mice (P<0.05 or P<0.01), and the spleen MDA content in group B was downregulated by sepia extract (P<0.01).

## 4. Discussion

Presently, chemotherapy is still the basic therapeutics course for tumour treatment. As a common chemotherapeutic, cyclophosphamine is an important clinical medicine. The drug can kill tumour cells and help patients to get well, but on the other hand, it is well known that the drug can also kill healthy cells of many tissues and organs. In this paper, we investigated the effects of cyclophosphamine on hemopoietic function, including organ index and antioxidant ability of spleen, bone marrow nucleated cells and peripheral blood profile.

As a part of hemopoietic system, the spleen participates in the regulatory mechanism of bone marrow hemopoiesis and removal of red blood cells and platelets. Organ index and antioxidant ability reflect its health status. In this investigation, it was found that cyclophosphamine significantly reduced the organ  index and antioxidant parameters of spleen, it is to say that cyclophosphamine caused injury to this organ. As a hemopoietic organ, the decreased quantity of bone marrow nucleated cells also indicated that the hemopoietic function of marrow was also affected by the chemotherapy. Additional evidence of hemopoietic injury of bone marrow induced by cyclophosphamine were the changes in the peripheral blood profile. This investigation proved that cyclophosphamine severely downregulated the amounts of erythrocytes, leukocytes and hemoglobin levels, but did not affect platelets. From above data we conclude that the hemopoietic function in mice was indeed damaged by cyclophosphamine, so in the course of clinical treatment of a tumour, well-balanced hemopoietic system may be subjected to  damage by the chemotherapy and accordingly, development of cell-protective drugs is both necessary and urgent.

The squid ink extract used in this work was crude and contained almost all the ingredients of squid ink, which has many kinds of components, such as melanin, proteins, lipids, glycosaminoglycans, etc,. except that the sand was removed. Background research has shown that squid ink possesses extensive biological roles. Lu *et al.* [6] reported that cuttlefish ink elevated leukocyte quantity to improve hemopoiesis of marrow in mice. The hemopoietic function of marrow relies on bone marrow nucleated cells, peripheral blood cell is from the proliferation and differentiation of hemopoietic stem cells and  progenitor cells, cell growth factors regulate together the proliferation and differentiation of hematopoietic stem cells. It has been proven that squid ink could induce many cytokines, such as IL-4 [17] and colony stimulating factor (CSF) [14]. CSF stimulates the proliferation and differentiation of hemopoietic stem cell and many kinds of progenitor cells, and increases the number of granulocyte and monocyte in blood as well as macrophage in tissue [18, 19]. To examine the protective effects of squid ink towards hemopoietic injury induced by chemotherapeutics, the marine substance was administered to model mice with damage induced by cyclophosphamine. We found that the natural product not only obviously affected the peripheral blood profile, such as erythrocytes, leukocytes and hemoglobin levels, and quantity of marrow nucleated cells, also changed the organ index and antioxidant ability of the spleen. The results indicate that squid ink extract can reverse the spleen damage and marrow hemopoiesis induced by cyclophosphamine and protect the body from chemotherapeutic injury. On the other hand, Lei *et al.* [20] and Pang *et al.* [21] showed that sepia could improve hemopoiesis of marrow in normal mice, however, this paper showed another different result, that is squid ink did not improve hemopoiesis of marrow in natural mice, and peripheral blood profile and number of bone marrow cells were not modified by the marine drug. Although squid ink extract had little effect on hemopoiesis of marrow in natural mice, elevation of antioxidant ability of spleen in natural mice was induced by the marine extract, which implied that squid ink may be conducive to improved hemopoietic function of natural mice by improving the function of spleen.

In addition, this paper provided other information. A low dosage of extract changed significantly the data (except for platelets), whereas, the high dosage products had negative effects, which implies that high dosage extract is not helpful but rather harmful to the body; it is necessary to further investigate the reasons for this, and also further investigation should be done to identify the optimum dosage of squid ink extract and confirm the effective component(s) of squid ink.

## References

[1] Palumbo A, Di Cosmo A, Gesualdo I, d’Ischia M (1997). A calcium dependent nitric oxide synthase and NMDA R1 glutamate receptor in the ink gland of Sepia officinalis: a hint to a regulatory role of nitric oxide in melanogenesis?. Biochem. Biophys. Res. Commun.

[2] Palumbo A, Poli A, Di Cosmo A, d’Ischia M (2000). N-Methyl-D-aspartate receptor stimulation activates tyrosinase and promotes melanin synthesis in the ink gland of the cuttlefish Sepia officinalis through the nitric oxide/cGMP signal transduction pathway. J. Biol. Chem.

[3] Palumbo A, Di Cosmo A, Poli A, Di Cristo C, d’Ischia M (1999). A calcium/calmodulin- dependent nitric oxide synthase, NMDAR2/3 receptor subunits, and glutamate in the CNS of the cuttlefish Sepia officinalis: localization in specific neural pathways controlling the inking system. J. Neurochem.

[4] Xie GL, He S (2002). Study of sepia improving natural killer cell activity in mice. J. Chin. Med. Univ.

[5] Takaya Y, Uchiswa H (1994). An investigation of the antitumor peptodoglycan fraction from aquid ink. Biol Pharm Bull.

[6] Lu T, Gao CY (1995). Effects of elevating leukocyte number of cuttlefish ink. Pract. J. Integ. Chin. Mod. Med.

[7] Sasaki J, Ishita K, Takaya Y, Uchiswa H, Matsue H (1997). Antitumor activity of squid ink. J. Nutr. Sci. Vitaminol.

[8] Lei M, Wang JF, Pang L, Wang YM, Chen SG, Xue CH (2007). Effects of sepia on the metabolization of blood lipid and antioxidant ability in hyperlipidemia rats. Chin. J. Mar. Drugs.

[9] Lei M, Wang JF, Wang YM, Pang L, Wang Y, Xu W, Xue CH (2007). Study of the radio-protective effect of cuttlefish ink on hemopoietic injury. Asia Pac. J. Clin. Nutr.

[10] Rajaganapathi J, Thyagarajam SP, Edward JK (2000). Study on cephalopod’s ink for anti-retroviral activity. Indian J. Exp. Biol.

[11] Takai M, Yamazaki K, Kawai Y, Inoue N, Shinano H (1993). Effects of squid liver, skin, and ink on chemical characteristics of “ika-shiokara” during ripening process. Bull. Jpn. Soc. Fish.

[12] Sadok S, Abdelmoulah A, Abed AE (2004). Combined effect of sepia soaking and temperature on the shelf life of peeled shrimp. Penaeus kerathurus Food Chem.

[13] Funatsu Y, Fukami K, Kondo H, Watabe S (2005). Improvement of “Kurozukuri Ika-Shiokara” (Fermented Squid Meat with Ink) odor with Staphylococcus Nepalensis isolated from the fish sauce mush of frigate mackerel Auxis Rochei. Bull Jpn Soc Fish.

[14] Xie GL, He S (2001). Study about CSF activity induced by sepia in mice. Chin. J. Mar. Drugs.

[15] Guan SB, Li YL, Guo YZ, Chen ZL, Zhan XM, Zhong JP, Li K, Liu HZ (2007). Protective effect of extract from ink of *lologo chinensis* on the marrow of mice. Strait Pharm. J.

[16] Zhang HQ, Lin AP, Sun Y, Deng YM (2001). Chemo-and radio-protective effects of polysaccharide of Spirulina platensis on hemopoietic system of mice and dogs. Acta Pharmaco. Sin.

[17] He S, Meng SN, Xie GL (2003). Study on secretion of interleukin-1 induced by cuttlefish ink in mice. Chin J Mar Drugs.

[18] Ganser A, Linelemannn A, Seipelt G, Ottmann OG, Herrmann F, Eder M, Frisch J (1991). Clinical effects of recombinant human interleukin-3. Am. J. Clin. Oncol.

[19] Guba SC, Sartor CI, Gottschalk LR, Ye-Hu J, Mulligan T, Emerson SG (1992). Bone marrow stromal fibroblasts secrete interleukin-6 and granulocyte-macrophage colony stimulating factor in the absence of inflammatory stimulation: Demonstration by serum-free bioassay, enzyme-linked immunosorbent assay, and reverse transcriptase polymerase chain reaction. Blood.

[20] Lei M, Wang JF, Pang L, Gao S, Wang Y, Xue CH (2006). Effects of sepia on hematopoietis stem cells, granulocyte and monocyte progenitor cells and peripheral WBC in mice. Chin. J. Mar. Drugs.

[21] Pang L, Wang JF, Lei M, Wang Y, Xue CH (2007). The experimental studies on the effects sepia products on hematopoiesis in mice. Acta Nutr Sin.

